# Vaccination against the brown stomach worm, *Teladorsagia circumcincta*, followed by parasite challenge, induces inconsistent modifications in gut microbiota composition of lambs

**DOI:** 10.1186/s13071-021-04688-4

**Published:** 2021-04-06

**Authors:** James Rooney, Alba Cortés, Riccardo Scotti, Daniel R. G. Price, Yvonne Bartley, Karen Fairlie-Clarke, Tom N. McNeilly, Alasdair J. Nisbet, Cinzia Cantacessi

**Affiliations:** 1 Department of Veterinary Medicine, University of Cambridge, Cambridge, UK; 2 Departament de Farmàcia I Tecnologia Farmacèutica I Parasitologia, Facultat de Farmàcia, Universitat de València, Valencia, Spain; 3 Vaccines and Diagnostics Department, Moredun Research Institute, Edinburgh, UK; 4 Disease Control Department, Moredun Research Institute, Edinburgh, UK

**Keywords:** *Teladorsagia circumcincta*, Gastrointestinal helminth, Ruminant, Microbiome, Vaccine, *Prevotella* spp.

## Abstract

**Background:**

Growing evidence points towards a role of gastrointestinal (GI) helminth parasites of ruminants in modifying the composition of the host gut flora, with likely repercussions on the pathophysiology of worm infection and disease, and on animal growth and productivity. However, a thorough understanding of the mechanisms governing helminth-microbiota interactions and of their impact on host health and welfare relies on reproducibility and replicability of findings. To this aim, in this study, we analysed quantitative and qualitative fluctuations in the faecal microbiota composition of lambs vaccinated against, and experimentally infected with, the parasitic GI nematode *Teladorsagia circumcincta* over the course of two separate trials performed over two consecutive years.

**Methods:**

Two trials were conducted under similar experimental conditions in 2017 and 2018, respectively. In each trial, lambs were randomly assigned to one of the following experimental groups: (i) vaccinated/infected, (ii) unvaccinated/infected and (iii) unvaccinated/uninfected. Faecal samples collected from individual animals were subjected to DNA extraction followed by high-throughput sequencing of the V3-V4 region of the bacterial 16S rRNA gene and bioinformatics and biostatistical analyses of sequence data.

**Results:**

Substantial differences in the populations of bacteria affected by immunisation against and infection by *T. circumcincta* were detected when comparing data from the two trials. Nevertheless, the abundance of *Prevotella* spp. was significantly linked to helminth infection in both trials.

**Conclusions:**

Despite the largely conflicting findings between the two trials, our data revealed that selected gut microbial populations are consistently affected by *T. circumcincta* infection and/or vaccination. Nevertheless, our study calls for caution when interpreting data generated from *in vivo* helminth-microbiome interaction studies that may be influenced by several intrinsic and extrinsic host-, parasite- and environment-related factors.
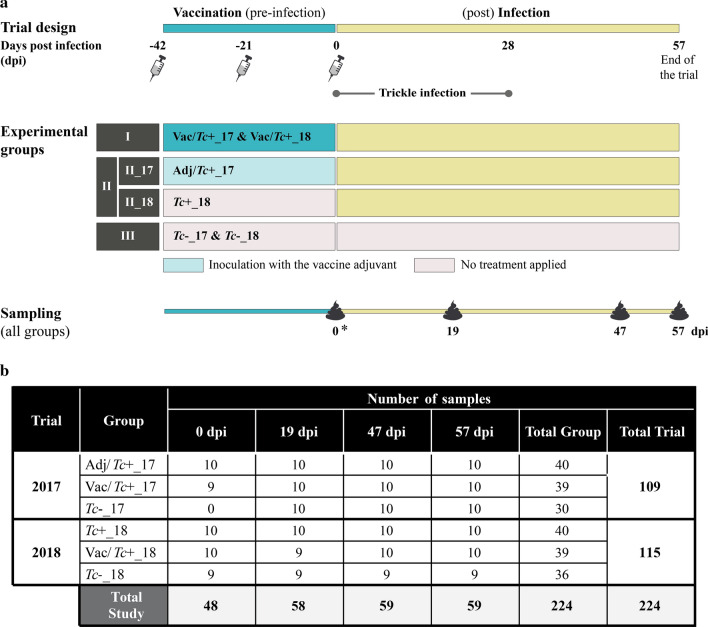

**Supplementary Information:**

The online version contains supplementary material available at 10.1186/s13071-021-04688-4.

## Background

It has been estimated that, by 2050, the global human population will exceed 9 billion and that such a dramatic growth will exacerbate the already strained ability to meet the increasing demand of meat and dairy products [1]; thus, to avoid a potential food security crisis, global efforts are directed towards improved livestock health management practices via disease prevention and control [2, 3]. Amongst infectious agents of livestock, parasitic helminths are a leading cause of disease in ruminants, with severe repercussions on both animal health and welfare, and production efficiency [3, 4]. Worldwide, such losses are estimated at US $3 billion annually because of the decreased feed conversion ratio, stock death and replacement and the costs linked to regular use of anthelmintics [5].

Whilst control of worms of livestock heavily relies on administration of antiparasitic drugs, global emergence of parasite strains resistant to all available classes of anthelmintics makes the use of parasiticides as a sole means of helminth control unsustainable. Thus, over the last decades, significant research efforts have been channelled into the development of vaccines against parasitic nematodes of ruminants [6–13], with several promising prototypes being developed [14–16]. Amongst these is a recombinant vaccine against one of the most economically important gastrointestinal (GI) helminths of small ruminants in temperate regions, *Teladorsagia circumcincta*, which resides in the abomasum or ‘true stomach’ [14, 17]. In particular, in a study by Nisbet et al. [14], a vaccine composed of eight recombinant helminth-derived proteins administered to sheep prior to challenge infection led to a reduction in *T. circumcincta* faecal egg counts (FEC) and abomasal worm numbers by up to 75% compared with non-vaccinated controls. The results of this and other studies [16, 18] show promise for the future use of vaccines as viable alternatives to anthelmintics for parasite eradication; nevertheless, vaccination studies published to date report varying efficacy, thus suggesting that other immunity-independent, unexplored factors may be contributing to parasite survival within the host [5, 18]. A deeper understanding of the fundamental biology of the parasite and of the interactions between worm, host and gut environment might assist to enhance vaccine efficacy and/or to develop novel effective means of parasite control.

Amongst these interactions, the cross-talk among the parasites, host and resident gut flora is receiving increasing attention, particularly due to the key roles of the vertebrate gut microbiome in immunity development and defence against pathogens [19–21]. Indeed, over the last few years, a growing number of studies have provided mounting evidence that GI helminth infections can significantly impact the composition of the ruminant gut microbiota [22–25]. The effects of worm-mediated changes in gut microbiota composition on the host ability to mount effective immune responses against the invading parasites are yet to be fully explored. Nevertheless, in a recent study [25], we investigated the fluctuations in faecal microbiota composition of lambs vaccinated against, and experimentally infected with, *T. circumcincta*; immunisation was achieved by inoculation of eight recombinant parasite antigens [25]. In this study, qualitative and quantitative changes in faecal microbiota composition were associated mainly with parasite infection rather than with vaccination-induced immunity. However, data reproducibility across independent studies and cohorts of helminth-infected animals is pivotal in order to harness the potential benefits of gut microbiome manipulation for parasite control [22, 26–28].

Recently, Nisbet et al. [16] compiled data from several independent *in vivo* trials to select a pair of recombinant immunogens from the original eight-component vaccine (i.e. a mutated form of a calcium-dependent apyrase [mTci-APY-1] and an astacin-like metalloprotease derived from *T. circumcincta* [Tci-MEP-1]) whose administration had the most impact of each of the original eight antigens on reducing cumulative faecal egg counts (cFEC) post-challenge infection. The present project builds on data from these trials, as well as on findings from Cortés et al. [25], to investigate reproducibility of worm-microbiota interaction studies using the *Teladorsagia*-sheep system. In particular, we utilise high-throughput amplicon sequencing of the bacterial 16S rRNA gene coupled with bioinformatics and biostatistical analyses of sequence data to explore the effect of vaccination against, and experimental infection with, *T. circumcincta* in two independent *in vivo* trials carried out over consecutive years under largely similar experimental conditions. We show that, whilst the full set of taxonomic changes in faecal microbiota composition of lambs infected with *T. circumcincta* is inconsistent between trials, the relative abundance of selected bacterial groups (e.g. *Prevotella*) are reproducibly altered upon colonisation by this parasite and might therefore represent biomarkers of infection.

## Materials and methods

### Experimental design, sample collection and molecular biology procedures

Faecal samples analysed in this study were derived from animals enrolled in two separate trials, conducted in 2017 [16] and 2018 (A.J. Nisbet, unpublished data), respectively. Briefly, in each trial, 30 Texel crossbred lambs, five to six months of age and helminth-free (verified and confirmed by parasitological examination of individual faecal samples prior to the beginning of the study), were randomly divided into three age- and gender-balanced groups of ten lambs each. Each group (group 1–3) was housed in a separate pen.

**Fig. 1:**
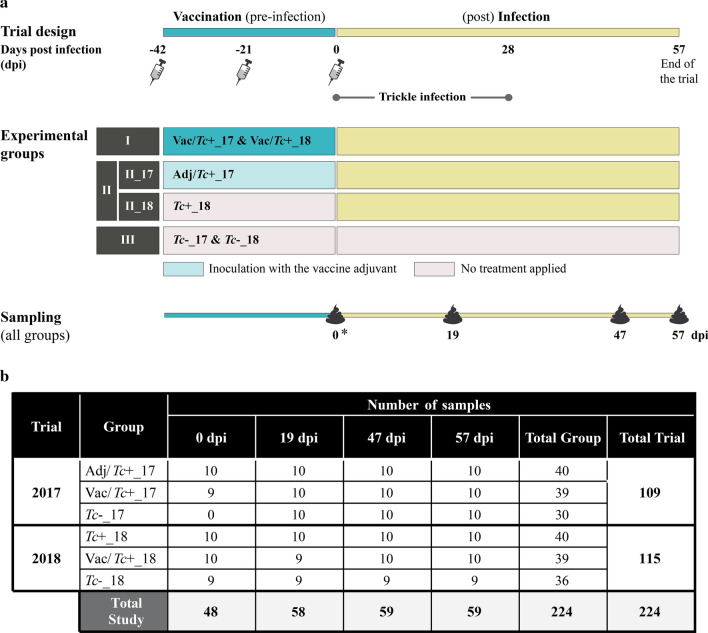
**a** Experimental design. Two studies aimed to investigate the impact of vaccination against, and experimental infection with, *Teladorsagia circumcincta* (*Tc*) were conducted in 2017 (17) and 2018 (18), respectively. In each experiment, three groups of lambs (I to III) were enrolled and subjected to (I) inoculation of a recombinant vaccine against *T. circumcincta* followed by experimental infection (Vac/*Tc*+_17 and Vac/*Tc*+_18, respectively); (II) experimental infection with or without prior inoculation of vaccine adjuvant (Adj/*Tc*+_17 and *Tc*+_18, respectively), while lambs in groups III were left unvaccinated and uninfected (*Tc*−_17 and *Tc*−_18, respectively). Faecal samples were collected at designated time points for DNA extraction and high-throughput sequencing of the bacterial 16S rRNA gene. *Samples for *Tc*−_17 were unavailable. **b** Number of faecal samples subjected to metagenomics sequencing, according to trial, animal group and time point

Lambs enrolled in the 2017 trial were randomly assigned to one of three groups: (i) lambs injected three times with a recombinant vaccine containing mTci-APY-1 and Tci-MEP-1, leaving a three-week interval between successive immunisations (‘Vac/*Tc*+_17′) (cf. [16]); (ii) lambs injected with the vaccine vehicle (i.e. urea, PBS and the adjuvant Quil A) (‘Adj/*Tc*+_17′); (iii) unimmunised and uninfected (‘*Tc*−_17′). Lambs enrolled in the 2018 experiment were (i) vaccinated with a recombinant vaccine as above (‘Vac/*Tc*+_18′), (ii) left unimmunised (‘*Tc*+_18′) or (iii) left unimmunised and uninfected (‘*Tc*−_18′) (Fig. 1). Following the final immunisation, each animal in groups ‘Vac/*Tc*+_17′, ‘Adj/*Tc*+_17′, ‘Vac/*Tc*+_18′ and ‘*Tc*+_18′ was experimentally infected with 2000 *T. circumcincta* infective larvae, administered orally three times per week for  four weeks (Fig. 1a). In both trials, lambs were separated into their treatment groups and groups were penned separately for  seven days prior to the first vaccination. All groups were housed in the same building (in adjacent pens), except for *Tc*−_18, which was housed in a separate building due to space constraints.

To estimate vaccine efficacy in each trial, FECs were performed twice a week using the salt flotation technique, sensitive to 1 egg per gram (EPG), as described by Nisbet et al. [16]. At the end of each trial, worms were enumerated from the abomasa of each infected lamb using standard techniques and cFECs were calculated using the trapezoidal method. Statistical analyses were performed as previously described in Nisbet et al. [16]. Briefly, the % reduction in cFEC following vaccination was calculated as follows:

$$\% {\text{ cFEC reduction }} = \, \left[ {{1} - \left( {{\text{cFECv}}/{\text{mean cFECc}}} \right)} \right] \, \times { 1}00$$

where cFECv = cFEC for an individual vaccinated animal in any given trial, and mean cFECc = average cFEC in control (i.e., unvaccinated and infected) animals in the same trial.

For faecal microbiome sequencing and characterisation, samples were collected directly from the rectum of each lamb at the end of the immunisation period (prior to experimental infection) (0 days post infection [dpi]) and at 19, 47 and 57 dpi for Vac/*Tc*+_17 and Adj/*Tc*+_17 (2017 trial), and Vac/*Tc*+_18, *Tc*+_18 and *Tc*−_18 (2018 trial). Samples were collected from lambs in the *Tc*−_17 group at all above-mentioned time points except at 0 dpi (Fig. 1). Faecal samples were snap frozen at – 80 °C and stored until DNA extractions and high-throughput sequencing of the V3-V4 region of the bacterial 16S rRNA gene, which were performed using the protocols described in Cortés et al*.* [25].

### Bioinformatics and statistical analyses of bacterial 16S rRNA gene sequence data

Sequence data were analysed using the Quantitative Insights Into Microbial Ecology 2 (QIIME2-2020.2; https://qiime2.org) software [29]. Each raw paired-end Illumina library was separately quality filtered, dereplicated and chimeras identified in QIIME2 using DADA2 [30]. High-quality library outputs were merged and sequences were clustered into Amplicon Sequence Variants (ASVs) on the basis of similarity to known bacterial sequences available in the SILVA database (v138; https://www.arb-silva.de/; 99% sequence similarity cut-off). The ASV table with the assigned taxonomy was exported from QIIME2 and statistical analyses were conducted using the Calypso software (cgenome.net/calypso/) [31]. Cumulative sum scaling (CSS) was applied, followed by log2 transformation to account for the non-normal distribution of taxonomic counts data. Initially, microbial profile data were clustered using Principal Coordinates Analysis (PCoA) with the Bray-Curtis distance metric. In addition, a supervised Canonical Correspondence Analysis (CCA) was performed, using ‘treatment/infection’ as explanatory variable. Faecal bacterial alpha diversity was calculated for each treatment/infection group using the Shannon Index, richness and evenness; within each group, differences in faecal bacterial alpha diversity over time were evaluated by Mixed Effect Linear Regression (MELR), whilst differences between groups at each time point were assessed by ANOVA. Faecal beta diversity for each group was calculated using Bray-Curtis dissimilarity. Differences in beta diversity over time within each group were calculated using Analysis of Similarity (ANOSIM) [32], whilst pairwise comparisons of microbial communities in samples collected at different time points were carried out using Permutational Multivariate Analysis of Variance (PERMANOVA) [33], using an additional plugin in QIIME2, i.e. the q2-diversity-plugin, which utilises the beta-group-significance function. Changes in beta diversity between groups were assessed at each time point by ANOSIM. Changes in the relative abundances of bacterial taxa over time within a given treatment group were measured using MELR, and False Discovery Rate (FDR) was applied to account for multiple comparisons. Within each animal group, pairwise differences in faecal microbial taxon abundances between time points were evaluated using Tukey’s test. Finally, the Linear Discriminant Analysis Effect Size workflow (LEfSe) [34] was used to calculate differences in the abundances of individual microbial taxa (phylum to genus) between groups at each time point.

## Results

### Vaccine efficacy

As described in Nisbet et al. [16], in the 2017 trial, lambs in all groups began to excrete *T. circumcincta* eggs from 16 to 19 dpi and, at peak egg shedding (day 40), mean FEC in the vaccine recipients (Vac/*Tc*+_17) was 186 ± 45 EPG *vs*. a mean FEC in the control lambs (Adj/*Tc*+_17) of 304 ± 75 EPG. A Generalised Additive Mixed Modelling (GAMM) analysis of the FEC data over time identified a difference between the mean FEC of the Vac/*Tc*+_17 group *vs.* the Adj/*Tc*+_17 group, although this did not reach statistical significance (*p* = 0.093). Despite a reduction in mean cFEC levels by 43% in the Vac/*Tc*+_17 group compared to the Adj/*Tc*+_17 group, no statistically significant differences between groups were observed (*p* = 0.079) (Additional file 1). Abomasal nematode burdens post mortem were reduced by 52% in vaccine recipients compared with control lambs which received adjuvant only; however, this difference was not statistically significant (*p* > 0.05) (Additional file 1).

In the 2018 trial, egg shedding at day 40 was 175 ± 47 EPG in vaccinated animals (Vac/*Tc*+_18) *vs.* 258 ± 75 EPG in unvaccinated, challenged animals (*Tc*+_18). Mean cFEC levels and abomasal nematode burdens at post mortem were reduced by 16% and 12% in vaccinated *vs.* unvaccinated challenged animals, respectively; however, these reductions were not statistically significant (*p* > 0.05) (Additional file 1).

### Overall faecal microbial profiles of lambs enrolled in the 2017 and 2018 trials

A total of 224 faecal samples were collected during the course of this study (i.e. 109 and 115 for the 2017 and 2018 trial, respectively) (cf. Fig. 1b); deep metagenomic amplicon sequencing of these samples yielded a total of 109,761,783 paired-end reads, of which 44,319,879 (77,890 per sample mean ± 51,806 standard deviation) were retained after quality filtering. These reads were subsequently assigned to 128,945 ASVs, 18 bacterial phyla and 1 archaeal phylum, respectively (Additional file 2). Raw sequence data are available from the European Nucleotide Archive (ENA) database under accession number PRJEB32873, whilst a summary of curated data can be accessed via MICHELINdb at www.helminthsandmicrobes.vet.cam.ac.uk [35].

**Fig. 2:**
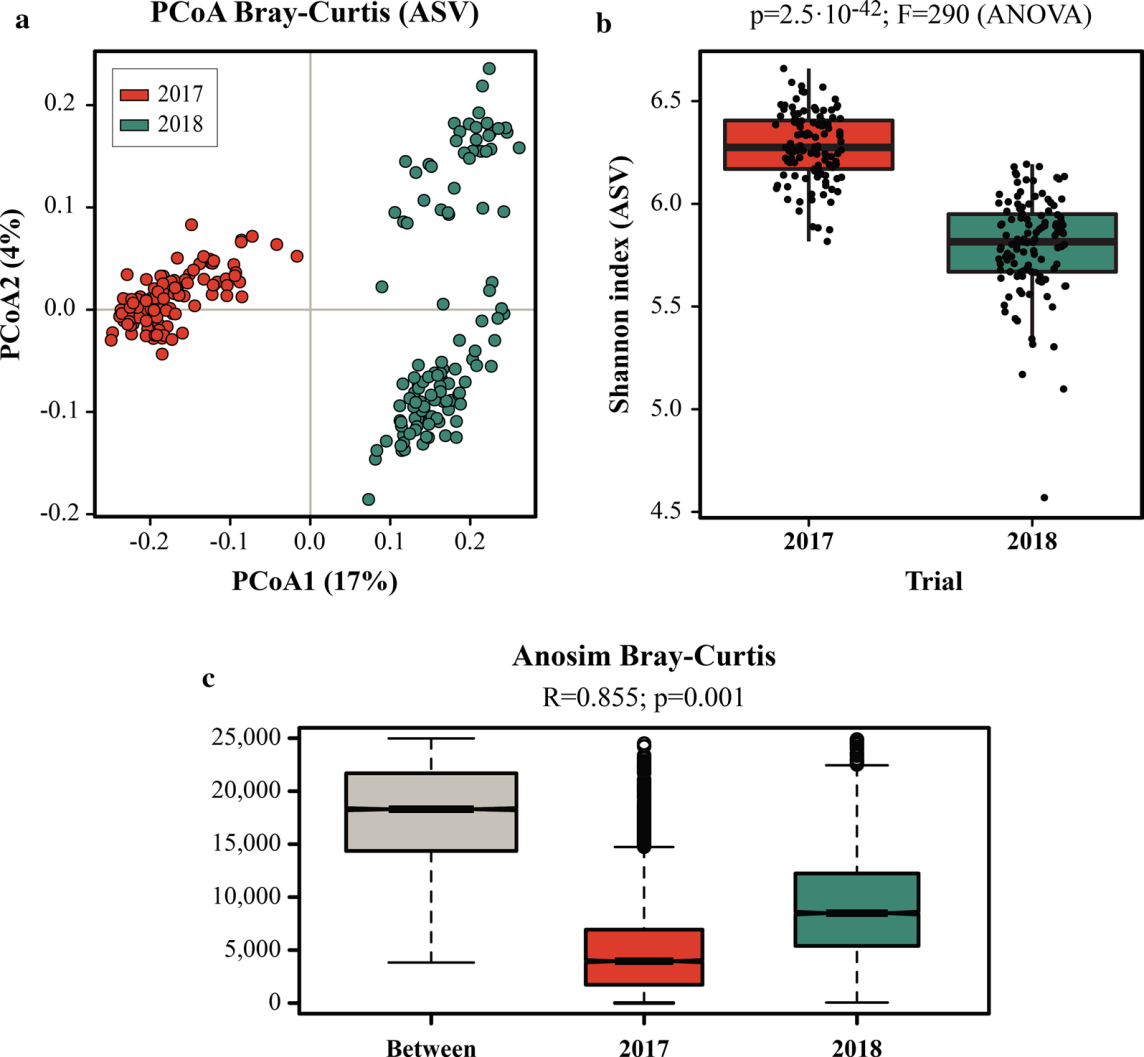
Comparison of microbial community profiles of faecal samples collected over the course of the 2017 and 2018 trials. Microbial profiles ordinated by Principal Coordinates Analysis (PCoA) (**a**) and differences in microbial alpha (**b**) and beta (**c**) diversity recorded between samples collected over the 2017 and 2018 trial, respectively.

The overall faecal microbial profiles of ruminant samples collected in the 2017 and 2018 trial are summarised in Additional file 2. The faecal microbial profiles of lambs enrolled in both trials were ordinated by PCoA, which revealed substantial differences between samples collected during the two trials (Fig. 2a). This observation was supported by statistically significant differences yielded by CCA analysis of 2017 *vs.* 2018 samples (*p* = 0.001, F = 26.86). Accordingly, significant differences in overall diversity metrics were observed between samples collected in the 2017 *vs.* 2018 trial, with the latter displaying significantly lower microbial alpha diversity and higher beta diversity than the former (Fig. 2b, c). Moreover, to identify key bacteria associated with the faecal microbiota of lambs enrolled in each trial, raw counts data of 2017 *vs.* 2018 were compared using Partial Least Squares Discriminant Analysis (sPLS-DA) built into the multivariate data analysis framework mixMC implemented in Calypso [36] (Additional file 3). Given the substantial differences in the overall microbial profiles of animals enrolled in each trial, subsequent tests, consisting of (i) analyses of longitudinal variations within each experimental group over the course of the experiment, along with (ii) cross-sectional comparisons between groups at each time point, were performed separately for 2017 and 2018 samples, respectively.

### Changes in gut microbial communities associated with vaccination against, and experimental infection with, *T. circumcincta*

**Fig. 3:**
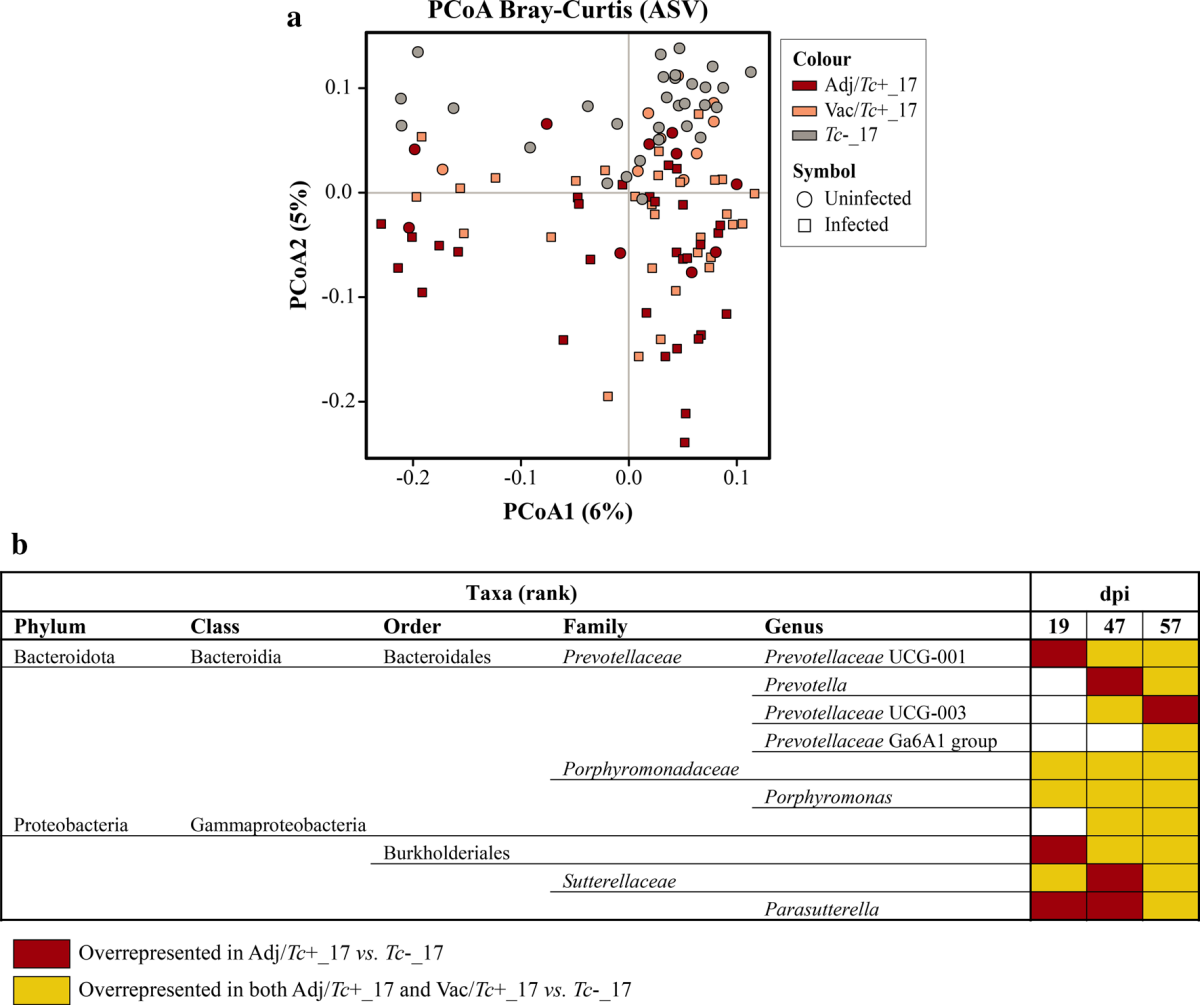
**a** Principal Coordinates Analysis (PCoA) of faecal microbial profiles of lambs experimentally infected with *Teladorsagia circumcincta* following adjuvant (Adj/*Tc*+_17) or vaccine (Vac/*Tc*+_17) administration and uninfected controls (*Tc*−_17) over the course of the 2017 trial. Microbial profiles were clustered by experimental group (colour) and infection status (symbol); for lambs in the Adj/*Tc*+_17 and Vac/*Tc*+_17 groups, uninfected samples correspond to those collected at 0 days post infection (dpi). **b** Selected microbial taxa displaying significantly higher abundance in faecal samples from experimentally infected lambs (i.e. Adj/*Tc*+_17 and/or Vac/*Tc*+_17) compared to uninfected controls. Results based on Linear Discriminant Analysis Effect Size (LEfSe); LDA score (log10) > 3

For samples collected in the 2017 trial, unsupervised PCoA revealed clustering of microbial profiles according to ‘infection status’ along Principal Coordinate 2. In particular, samples from the *Tc*−_17 group clustered together with samples from both Adj/*Tc*+_17 and Vac*/Tc+_*17 groups at 0 dpi and diverged from Adj/*Tc*+_17 and Vac*/Tc+_*17 samples collected post-infection, albeit with some overlap (Fig. 3a). Whilst no significant fluctuations in microbial alpha diversity (Shannon index) were detected within each group over time (Additional file 4), changes in gut microbial beta diversity were observed within each group throughout the course of the experiment; nevertheless, these changes did not reflect a solid trend associated with infection and/or vaccination over time (Additional file 4).

Changes in the abundances of individual bacterial taxa in faecal samples collected from each experimental group over time were assessed by MELR [FDR-adjusted *q* < 0.05] (Additional file 5). Amongst others, a significant reduction in *Akkermansiaceae* and the genus *Akkermansia* was observed in samples collected from Adj/*Tc*+_17 and Vac/*Tc*+_17 between 0 and 19 dpi, which was retained until the end of the trial (Additional file 5). In addition, significant increases in the abundance of bacteria belonging to the family *Prevotellaceae*, including the genera *Prevotella*, *Prevotellaceae* UCG-001 and *Prevotellaceae* UCG-003, were detected in the faecal microbiota of both Adj/*Tc*+_17 and Vac/*Tc*+_17 over time (Additional file 5).

Changes in the abundance of selected bacterial groups were detected solely in either the Adj/*Tc*+_17 or Vac/*Tc*+_17 over time (Additional file 5); amongst these, bacteria belonging to the family *Porphyromonadaceae*, genus *Porphyromonas* and order *Burkholderiales*, family *Sutterellaceae*, were progressively expanded over time in the former group (Additional file 5). However, following pairwise post hoc testing by Tukey’s test, statistically significant differences in the abundance of *Porphyromonas* and *Porphyromonadaceae* were only achieved between 0 and 57 dpi, while no significant differences were recorded in the abundance of *Burkholderiales* or *Sutterellaceae* between any time point pairs (Additional file 5). None of the abovementioned taxa changed significantly in abundance in faecal samples from *Tc*−_17 over the course of the trial (Additional file 5).

The impact of vaccination and/or experimental infection on the ovine gut microbiota composition was further analysed cross-sectionally by comparing samples collected from every experimental group at each time point. The microbial profiles of samples collected throughout the 2017 trial were ordinated by CCA according to experimental group; notably, significant differences were observed between Adj/*Tc*+_17, Vac/*Tc*+_17 and *Tc*−_17 from 19 dpi onwards (Additional file 6). No differences in faecal microbial alpha diversity were detected between group pairs at any time point (Additional file 7); however, significant differences in faecal bacterial beta diversity were observed between *Tc−*_17 and Adj/*Tc*+_17, *Tc−*_17 and Vac/*Tc*+_17, and Adj/*Tc*+_17 and Vac/*Tc*+_17 at all post-infection time points, with the highest beta diversity detected in faecal samples from Adj/*Tc*+_17 animals (Additional file 7).

Differences in the abundances of individual faecal bacterial taxa (phylum to genus) between each pair of experimental groups at each time point were assessed by LEfSe [LDA score (log10) > 2.5] (Additional file 8). Amongst others, *Porphyromonas* (and family *Porphyromonadaceae*) were significantly increased in samples from either Adj/*Tc*+_17 or Vac/*Tc*+_17 compared with *Tc*−_17 samples from 19 dpi onwards, alongside the genera *Prevotellaceae* UCG-001, *Prevotella* and *Prevotellaceae* UCG-003 at 47 and/or 57 dpi (Fig. 3b and Additional file 8). *Parasutterella* (genus to phylum) was overrepresented in Adj/*Tc*+_17 compared to *Tc*−_17 at all post-infection time points and in Vac/*Tc*+_17 at 57 dpi (Fig. 3b and Additional file 8). Furthermore, the genus *Akkermansia* was significantly reduced in both infected groups compared to uninfected animals at 57 dpi (Additional file 8).

**Fig. 4:**
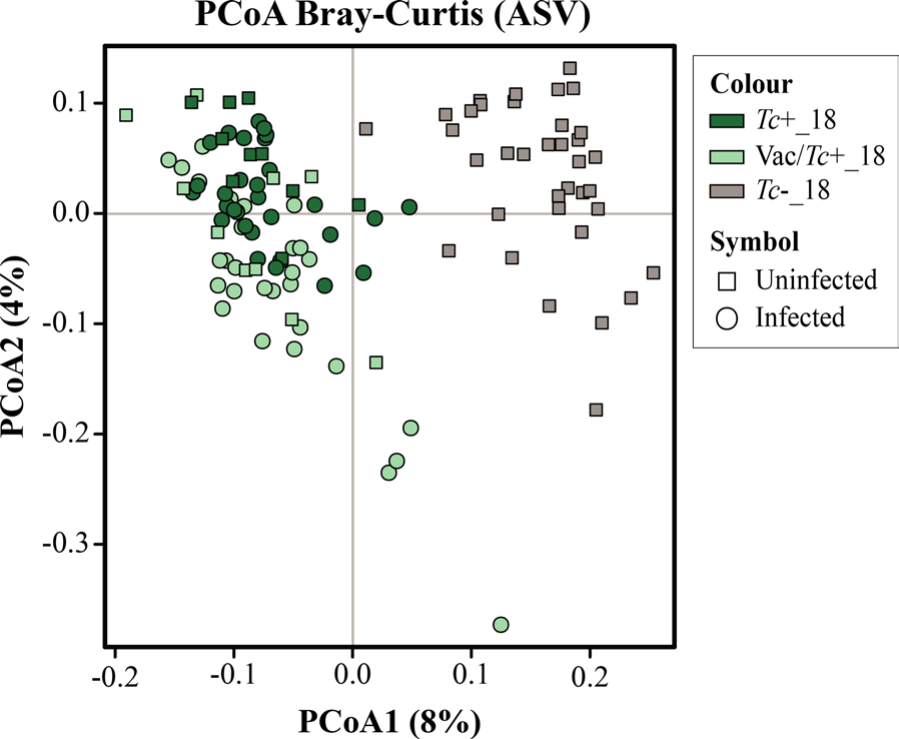
Principal coordinates analysis (PCoA) applied to the faecal microbiota of lambs infected with *Teladorsagia circumcincta*, solely (*Tc*+_18) or following vaccine administration (Vac/*Tc*+_18), and uninfected controls (*Tc*−_18) over the 2018 trial. Samples were clustered by experimental group (colour) and infection status (symbol); uninfected samples of *Tc*+_18 and Vac/*Tc*+_18 correspond to those collected at 0 days post infection (dpi)

For the 2018 trial, PCoA analysis of microbial profiles of faecal samples collected at all time points revealed clustering according to experimental group along Principal Coordinate 1, with samples from *Tc*+_18 and Vac/*Tc*+_18 (which included samples collected prior to experimental infection) clustering together, and to the exclusion of samples from the *Tc*−_18 group (Fig. 4). This dissimilarity was suggestive of profound differences in the baseline gut microbiota composition of *Tc*+_18 and Vac/*Tc*+_18 *vs.* *Tc*−_18 animals, which prevented us from conducting direct pairwise comparisons of microbial taxa abundances between groups. To identify microbial taxa accountable for this dissimilarity, raw counts data of *Tc*+_18 *vs.* *Tc*−_18 and Vac/*Tc*+_18 *vs.* *Tc*−_18 at baseline (i.e. 0 dpi) were compared by sPLS-DA. Using this approach, the genera *Lachnospiraceae* NK4A136 group*, Ruminococcus, Bacteroides, Oscillibacter,* UCG-002 (family *Oscillospiraceae*), *Eubacterium siraeum* group*, Phascolarctobacterium, Clostridia* UCG-014 and *RF39* were identified as discriminatory between *Tc*−_18 and both *Tc*+_18 and Vac/*Tc*+_18 (Additional file 9). Furthermore, analysis of alpha diversity revealed significantly lower Shannon index and microbial richness in samples from *Tc*−_18 compared to *Tc*+_18 and Vac/*Tc*+_18 at baseline (Additional file 10). Thus, further analyses aimed to identify gut bacterial taxa associated with *T. circumcincta* infection were performed as for samples collected in 2017 (i.e. by a combination of longitudinal and cross-sectional approaches), albeit taking into account baseline differences between the gut microbiota composition of *Tc*+_18 and Vac/*Tc*+_18 animals and that of *Tc*−_18.

No significant changes in gut microbial alpha diversity (Shannon index) were recorded for *Tc*+_18, Vac/*Tc*+_18 or *Tc*−_18 over time (Additional file 11). In contrast, microbial beta diversity was significantly increased in each group between 0 and 57 dpi (Additional file 11), and variations in the abundances of selected microbial taxa were observed within each experimental group over time (Additional file 12). Nonetheless, only a small number of low-abundant taxa (< 1% in relative abundance) displayed infection- and/or vaccination-associated variations that were sustained over the course of the trial. For instance, the genera *Romboutsia* and *M2PB4-65* termite group were significantly expanded over time in the *Tc*+_18 and Vac/*Tc*+_18 groups, whereas the genus *Victivallis* decreased in abundance over the course of the trial; however, these changes detected by MELR (FDR-adjusted *q* < 0.05) were not consistently supported by pairwise post hoc analysis (i.e. Tukey’s test) (Additional file 12). Additionally, the faecal microbiota of *Tc*+_18 animals was characterised by significant increases of the order *Burkholderiales*, genera *Prevotellaceae* UCG-003 and *Parasutterella* over the course of the experiment (albeit for *Burkholderiales* and *Parasutterella* no statistically significant differences between pairs of time points were detected by *post hoc* analysis). In contrast, the abundance of *Bacteroidales* BS11 gut group was significantly increased in Vac/*Tc*+_18 animals over time, with significant differences detected between 0 and 57 dpi by Tukey’s test (Additional file 12). Furthermore, the bacterial family *Porphyromonadaceae* and genus *Porphyromonas* were significantly reduced within the Vac/*Tc*+_18 group from 19 dpi onwards, albeit with significant differences detected by Tukey’s test only at the family level and between 19 and 47 dpi (Additional file 12).

The gut microbial profiles of each *Tc*+_18 and Vac/*Tc*+_18 were ordinated by CCA, following removal of samples from *Tc*−_18 due to the aforementioned substantial differences in baseline microbial profiles (cf. Fig. 4). Significant differences were detected between the overall microbial profiles of each pair of experimental groups at each time point (Additional file 13). Whilst no differences in faecal microbial alpha diversity were detected between group pairs at any post-infection time point (Additional file 14), analysis of beta diversity revealed significant differences between each pair of experimental groups at all time points, albeit with no consistent pattern (Additional file 14).

The complete list of faecal bacterial taxa (phylum to genus) whose abundances differed between each pair of experimental groups at each time point [as assessed by LEfSe, LDA score (log10) > 2.5] is available from Additional file 15. These included the genus and family *Bacteroidales* BS11gut group, which was significantly expanded in samples collected from Vac/*Tc*+_18 at each time point compared to both *Tc*+_18 and *Tc*−_18. Following independent analyses of the full set of taxonomic alterations occurring in the faecal microbial composition of lambs in each vaccination trial, it is worth noting that none of the microbial taxa associated with *T. circumcincta* infection in either 2017 or 2018 were included amongst the taxa that accounted for the main baseline differences in microbiota composition between animals enrolled in each trial by sPLS-DA (cf. Additional file 3).

### *Prevotella* is consistently associated with *T. circumcincta* infection

Given the fundamental differences in gut microbiota composition between animals enrolled in the 2017 and 2018 trials (both at baseline and over the course of each experiment), the LEfSe workflow was applied to the identification of faecal microbial genera associated with infection with, and/or vaccination against, *T. circumcincta* in both trials, whilst controlling for inter-experimental variability. In particular, the microbial taxa associated with *T. circumcincta* infection were identified by applying LEfSe to all samples collected post-experimental infection *vs.* samples from uninfected animals and samples collected pre-experimental infection from both the 2017 and 2018 trials. The genera *Prevotella* and *Lachnospiraceae NC2004* group were associated with infected samples, while the genus *Turicibacter* was linked to samples from uninfected lambs [LDA score (log10) > 3]. To account for potential biases related to animal growth, the same test was applied to samples collected from *Tc*−_18 animals at 0 dpi *vs.* 19 + 47 + 57 dpi [no samples were available for *Tc*−_17 at 0 dpi (cf. Fig. 1a)]. The results revealed that the genus *Lachnospiraceae NC2004* group was significantly associated with samples collected at 19 + 47 + 57 dpi in *Tc*−_18 animals (Additional file 16).

In addition, with the aim to identify faecal bacterial taxa potentially associated with vaccination against *T. circumcincta* and thus assess whether active immunisation might affect the response of the ovine gut microbiota to nematode infection, LEfSe was applied to samples from (unvaccinated) infected *vs.* vaccinated/infected animals of both trials. Whilst no taxa showed positive associations with vaccination, the genus *Prevotella* was significantly linked to unvaccinated/infected animals [LDA score (log10) > 3]*.*

## Discussion

Over the last decade, strong clues have emerged of a likely role of helminth-microbiota cross-talk in several mechanisms of host-parasite interactions, including processes of parasite infection and establishment, pathophysiology of helminth disease and modulation of host immune responses [22–25]. However, assessing the variability of parasite-associated quantitative and/or qualitative fluctuations in gut microbiota composition across studies and experimental systems is necessary for these interactions to be exploited for the development of novel strategies of parasite treatment and control [28]. In this study, we undertook analyses of changes in faecal microbiota composition of lambs vaccinated against, and infected with, *T. circumcincta* over two separate studies conducted in consecutive years. Several inconsistencies were detected between bacterial taxa associated with infection and vaccination in the 2017 and 2018 trial, in line with the known challenges linked to the reproducibility of helminth-microbiome interaction data and vaccination studies *in vivo* (reviewed by [28]). In ruminants, such variations might be linked to several factors including environmental conditions of temperature and humidity [37], and diet [38–42]. Whilst lambs enrolled in the 2017 and 2018 were fed an identical concentrate, minor differences, for example in hay constituents to which lambs had ad libitum access to and climatic conditions over the course of the two trials, might have resulted in substantial discrepancies in the baseline gut microbiota composition of these groups of animals. For instance, higher temperatures and raised relative humidity are associated with reduced feed intake that, in turn, affects the microbial composition of the ruminant gut [37–42]. Such variations are difficult to control in *in vivo* studies conducted in ruminant livestock; nevertheless, data from these experiments are pivotal to gain a deeper understanding of helminth-microbiota interactions in ‘real-world’ scenarios.

Despite substantial differences in the gut microbial make-up of lambs infected with, and vaccinated against, *T. circumcincta* across trials, fluctuations in microbial alpha- and beta diversity were similar over the course of the two experiments. In particular, no significant changes in faecal microbial alpha diversity were detected in either 2017 and 2018; this observation is in accordance with results from our previous study in *T. circumcincta*-infected sheep [25] as well as in goats infected with the ‘barber’s pole worm’ *Haemonchus contortus* [23] and cattle infected with the ‘brown stomach worm’ *Ostertagia ostertagi* [22], both of which are also located within the abomasum. Conversely, microbial beta diversity was increased in *T. circumcincta*-infected animals (i.e. Vac/*Tc*+_17 and Adj/*Tc*+_17) and in all groups (i.e. Vac/*Tc*+_18, *Tc*+_18 and *Tc−*_18) over the course of the 2017 and 2018 trial, respectively, albeit with no consistent trend between consecutive time points. However, given the increase in microbial beta diversity observed in unvaccinated/uninfected lambs over time in the 2018 study, the hypothesis that such a finding might result from natural variations in gut microbiome composition occurring in early life cannot be excluded [43, 44].

Amongst the gut bacteria whose abundances varied significantly following *T. circumcincta* infection in the 2017 study, the genus *Akkermansia* was reduced in faecal samples from Vac/*Tc*+_17 and Adj/*Tc*+_17 animals. Reduced populations of *Akkermansia,* a known mucin degrader, have been linked to deterioration of GI health in humans (reviewed by [45]). Despite the substantial compositional and functional differences between the gut microbiota of humans and ruminants, a study conducted by Chang et al. [46] demonstrated that *Akkermansia* was severely reduced in the microbiota of goats fed a high-concentrate diet (known to evoke an inflammatory response via the onset of subacute ruminal acidosis) in comparison to goats fed a low-concentrate diet. In addition, Scott et al*.* [47] demonstrated a significant loss of mucins in the abomasum of *T. circumincta*-parasitised sheep; this finding is consistent with a reduction in Muc5AC gene expression observed in sheep challenged with *H. contortus* [48]. Thus, the contraction of populations of *Akkermansia* observed in our study might be directly linked to a reduction of Muc5AC-encoded mucin, a predominant component of abomasal mucus.

Several bacterial taxa were positively associated with *T. circumcincta* infection in lambs enrolled in the 2018 study. Amongst these, the genus *Romboutsia* was significantly expanded over time in the *Tc*+_18 and Vac/*Tc*+_18 groups, in accordance with data from our previously published trial [25]. The genus *Romboutsia* has recently been linked to a broad range of metabolic functions, including carbohydrate utilization and fermentation of single amino acids [49]. Whilst information on the specific functions of *Romboutsia* in ruminants is scant, a single report described expanded populations of this genus of bacteria in the gut microbiota of diarrheic goats compared to healthy controls [50]. However, a clear association between abundance of *Romboutsia* in the gut microbiota of small ruminants and GI helminth infections is yet to be established.

Despite the fundamental differences in faecal bacterial taxa expanded upon *T. circumcincta* infection between the two studies, a small number of bacterial groups was consistently affected by helminth infection in both experiments. Amongst these, the order *Burkholderiales* and its genus *Parasutterella* were significantly expanded in faecal samples from Adj/*Tc*+_17 and *Tc*+_18. A bacterial genus closely related to *Parasutterella*, i.e. *Sutterella*, was also expanded in *T. circumcincta*-infected sheep enrolled in our previously published study [25]. Whilst this subtle difference is likely attributable to minor technical differences between data analysis software (i.e. QIIME2-2018.6 *vs* QIIME2-2020.2 and SILVA v.132 *vs* v.138) [25], the expansion of *Sutterella/Parasutterella* in *T. circumcincta*-infected animals might contribute to abomasal inflammation during worm establishment [51–53]; nevertheless, the exact mechanism(s) that determine the pro-inflammatory properties of these bacterial groups are yet to be fully elucidated [54, 55]. In addition, interestingly, the abomasal microbiota of mixed-breed sheep susceptible to infection by *H. contortus* was enriched in *Burkholderiales* compared with that of resistant animals [56]. However, it must be pointed out that the study by Tirabassi et al*.* [56] was characterised by a small sample size and relatively low metagenomic sequencing depth; in addition, a high proportion of bacterial taxa could only be annotated at the class level [56]. Thus, the identification of genus- and/or species-level *Burkholderiales* that contribute to helminth-associated proinflammatory responses, as well as whether expansion of these bacteria represent cause or consequence of worm establishment, remain to be clarified. Additionally, the *Bacteroidales *BS11 gut group genus was expanded in the Vac/*Tc*+_17 and Vac/*Tc*+_18 groups. In our previous study [25], we recorded significant increases of the *Bacteroidales *BS11 gut group family in faecal samples from both vaccinated/infected and unvaccinated/infected sheep. Bacteria from the *Bacteroidales *BS11 gut group are known producers of short-chain fatty acids (SCFAs), which play key roles in gut homeostasis, strengthening of the gut barrier and protection against inflammation [57, 58]. Zaiss et al. [57] and Li et al*.* [23] have demonstrated the ability of helminth infections to drive the expansion of populations of SCFA-producing gut bacteria, thus modulating host immune responses. To this end, previous exposure to *T. circumcincta*-derived molecules via vaccination might contribute to further expansion of *Bacteroidales *BS11 gut group bacteria, a hypothesis that requires thorough testing.

Given the substantial differences between findings from the 2017 and 2018 study, the LEfSe workflow was applied to the identification of taxa consistently associated with infection by, or vaccination against, *T. circumcincta*. Of these, the genus *Prevotella* was significantly linked to helminth infection when controlling for differences between trials. This observation is in accordance with data from our previous investigation [25], as well as from a study of the gut microbiota composition of *H. contortus*-infected goats [23]. Nevertheless, the biological significance of this change is yet to be determined and, whilst it has been hypothesised that the increased abundance of *Prevotella* might serve to counteract infection-induced protein loss [23], Cortés et al. [25] suggested that, under particular environmental conditions, this taxon might become a proinflammatory pathobiont, thus contributing to the pathophysiology of *Teladorsagia* infection (cf. [25]). Interestingly, amongst infected lambs, *Prevotella* was significantly associated with unvaccinated animals; whilst it must be noted that, unlike in our previous study [25], the effects of vaccine administration were minimal in both the 2017 and 2018 trial, it is tempting to speculate that vaccination against *T. circumcincta* might prevent a significant expansion of populations of *Prevotella *via the establishment of lower infection burdens compared to unvaccinated animals. These data support the hypothesis that, although infection is a major driver of microbial composition remodelling, vaccination may further reshape the ruminant microbial gut composition by reducing worm survival.

## Conclusions and future directions

Despite the largely conflicting findings between the two trials, our data revealed that selected gut microbial populations are consistently affected by *T. circumcincta* infection and/or vaccination. Nevertheless, our findings call for caution when interpreting data generated from *in vivo* helminth-microbiome interaction studies that may be influenced, amongst others, by factors including sampling site (e.g. mucosally associated *vs.* faecal microbiota), parasite infective dose, sampling protocols, environmental conditions and diet [28, 37, 40, 42, 59]. In particular, whilst *in vivo* studies of the vertebrate gut microbiome often rely on analyses of faecal samples for both ethical and practical reasons, qualitative and quantitative changes in selected populations of low-abundant bacteria inhabiting proximal sections of the GI system (e.g. the abomasum) might remain undetected and thus overlooked. For small ruminants, the use of *in vitro* systems (anaerobic incubation of ruminant gut fluids under controlled laboratory conditions (cf. [60]) might allow overcoming some of these limitations. Minimising the impact of these confounding factors is indeed key to the elucidation of the causality of worm-bacteria interactions and, in turn, to achieve a better understanding of whether, in the future, the manipulation of this cross-talk might be exploited in the development of novel parasite treatment and control strategies. In addition, the application of shotgun metagenomics sequencing methods, besides providing insights into the role(s) of viruses and eukaryotic microbes in helminth-microbiome cross-talk, may allow to characterise the putative functional implications of parasite-mediated changes in gut microbiome composition, and thus, the impact of such alterations on host health, welfare and productivity.

## Supplementary Information

**Additional file 1.** Mean (± standard error) values of (A) abomasal nematode burdens and cumulative faecal egg counts (cFEC) recovered from lambs infected with *Teladorsagia circumcincta* over the 2017 and 2018 trials, with (Vac/*Tc*+_17 and Vac/*Tc*+_18) or without (Adj/*Tc*+_17 and *Tc*+_18) prior immunisation.

**Additional file 2.** Gut microbial profiles of lambs enrolled in the 2017 and 2018 trial, respectively, at phylum (A), family (B) and genus (C) level. Plots display the mean relative abundances (calculated by total sum normalisation, i.e. TSS) of each taxon, whilst tables show mean ± standard deviation (SD). ‘Others’ includes all taxa representing < 0.5% (A) or < 1% (B and C) of the whole bacterial population.

**Additional file 3**. (A) Sparse Partial Least Squares (sPLS) regression applied to the faecal microbiota of lambs enrolled in each 2017 and 2018 trial. (B) Top bacterial genera discriminating the faecal microbiota of samples collected in each trial, identified by sPLS-Discriminant Analysis (sPLS-DA).

**Additional file 4**. Longitudinal changes in faecal microbial diversity of lambs experimentally infected with *Teladorsagia circumcincta* following adjuvant (Adj/*Tc*+_17) or vaccine (Vac/*Tc*+_17) administration, and of uninfected controls (*Tc*-_17), over the course of the 2017 trial. (A) Shannon index for alpha diversity; differences between time points were calculated by Mixed Effect Linear Regression (MELR). (B) ANOSIM plots depicting fluctuations in beta diversity over the course of the trial. Horizontal lines and asterisks indicate statistically significant differences between pairs of time points, calculated by permutational multivariate analysis of variance (PERMANOVA): *q < 0.05; **q < 0.01. ns: no sample available.

**Additional file 5**. Longitudinal changes in differentially abundant microbial taxa (FDR-adjusted *q* < 0.05) in faeces of unimmunised uninfected lambs enrolled in the 2017 trial.

**Additional file 6**. Faecal microbial profiles of lambs enrolled in the 2017 trial and infected with *Teladorsagia circumcincta *following adjuvant inoculation (Adj/*Tc*+_17) or immunisation (Vac/*Tc*+_17), as well as uninfected controls (*Tc*-_17), ordinated by Canonical Correspondence Analysis (CCA). Statistical differences between the microbial profiles of each experimental group at each time point post-trickle infection (dpi) are indicated at the top of each plot, whereas asterisks represent statistically significant differences between group pairs: **p < 0.01; ***p < 0.001.

**Additional file 7**. Differences in microbial alpha (A) and beta (B) diversity between experimental groups of lambs enrolled in the 2017 trial. (A) Shannon index (at Amplicon Sequence Variant level, ASV) calculated for each experimental group at each time point (dpi) and statistical differences between groups were assessed by ANOVA. (B) Overall and pairwise differences in Bray-Curtis dissimilarity between experimental groups were calculated by ANOSIM at each time point: **p < 0.01; ***p < 0.001. Adj/*Tc*+_17: lambs infected with *Teladorsagia circumcincta* following inoculation of the vaccine adjuvant; Vac/*Tc*+_17: lambs immunised against and subsequently experimentally infected with *T. circumcincta*; *Tc*-_17: uninfected lambs; ns: no sample available.

**Additional file 8**. Differentially abudant microbial taxa (CSS+log) in faecal samples of lambs experimentally infected with *Teladorsagia circumcincta* following adjuvant (Adj/*Tc*+_17) or vaccine (Vac/*Tc*+_17) administration *vs.* uninfected controls (*Tc*-_17) (2017 trial). Results based on Linear Discriminant Analysis Effect Size (LEfSe)—LDA score (log10) > 2.5.

**Additional file 9**. Sparse Partial Least Squares Discriminant Analysis (sPLS-DA) applied to the faecal microbiota of lambs enrolled in the 2018 trial at 0 days post-trickle infection, clustered by experimental group. (A) Comparison between infected (*Tc*+_18) *vs.* uninfected (*Tc*-_18) animals. (B) Comparison between vaccinated (Vac/*Tc*+_18) *vs.* uninfected (*Tc*-_18) animals. For each group pair compared, sPLS regression (left) and top bacterial genera discriminating the faecal microbiota of samples collected from each experimental animal, identified by sPLS-Discriminant Analysis (sPLS-DA).

**Additional file 10**. Differences in microbial alpha diversity between faecal samples of lambs infected with *Teladorsagia circumcincta*, either with (Vac/*Tc*+_18) or without prior immunisation (*Tc*+_18), and of uninfected controls (*Tc*-_18), enrolled in the 2018 trial. Horizontal lines indicate differences between time points: **p < 0.01; ***p < 0.001.

**Additional file 11**. Longitudinal changes in faecal microbial diversity of lambs enrolled in the 2018 trial and infected with *Teladorsagia circumcincta*, either with (Vac/*Tc*+_18) or without prior immunisation (Vac/*Tc*+_18), and of uninfected controls (*Tc*-_18). (A) Shannon index for alpha diversity; differences between time points were calculated by Mixed Effect Linear Regression (MELR). (B) ANOSIM plots depicting fluctuations in beta diversity over the course of the trial. Horizontal lines and asterisks indicate statistically significant differences between pairs of time points, calculated by permutational multivariate analysis of variance (PERMANOVA): *q < 0.05; **q< 0.01.

**Additional file 12**. Longitudinal changes in differentially abundant microbial taxa (FDR-adjusted *q* < 0.05) in faeces of unimmunised uninfected lambs enrolled in the 2018 trial.

**Additional file 13**. Faecal microbial profiles of lambs enrolled in the 2018 trial and infected with *Teladorsagia circumcincta*, either without (*Tc*+_18) or following prior immunisation (Vac/*Tc*+_18), as well as uninfected controls (*Tc*-_18), ordinated by Canonical Correspondence Analysis (CCA). Statistical differences between the microbial profiles of each experimental group at each time point post-trickle infection (dpi) are indicated at the top of each plot, whereas asterisks represent statistically significant differences between group pairs: **p < 0.01; ***p < 0.001.

**Additional file 14**. Differences in faecal microbial alpha (A) and beta (B) diversity between groups of lambs enrolled in the 2018 trial. (A) Shannon index (at Amplicon Sequence Variant level, ASV) calculated for each experimental group at each time point and statistically significant differences between groups assessed by ANOVA. (B) Overall and pairwise differences in Bray-Curtis dissimilarity between experimental groups, calculated by ANOSIM at each time point: *p < 0.05; **p< 0.01; ***p < 0.001. *Tc*+_18: lambs infected with *Teladorsagia circumcincta*; Vac/*Tc*+_18: lambs vaccinated against and subsequently infected with *T. circumcincta*; *Tc*-_2018: uninfected lambs.

**Additional file 15**. Differentially abundant microbial taxa (CSS+log) between faecal samples of lambs experimentally infected with *Teladorsagia circumcincta*, solely (*Tc*+_18) or following experimental vaccination (Vac/*Tc*+_18), *vs.* uninfected controls (*Tc*-_18) (2018 trial). Results based on Linear discriminant analysis Effect Size (LEfSe)—LDA score (log10) > 2.5.

**Additional file 16**. Differentially abundant faecal microbial genera (CSS+log) between samples collected at 0 and +19, +47 and +57 days post-infection (dpi) from uninfected animals in the 2018 trial (*Tc*-_18). Differences assessed by Linear discriminant analysis Effect Size (LEfSe)—LDA score (log10) > 2.5.

## Data Availability

Raw sequence data are available from the European Nucleotide Archive (ENA) database under accession number PRJEB32873, whilst a summary of curated data can be accessed via MICHELINdb at www.helminthsandmicrobes.vet.cam.ac.uk

## Abbreviations

ANOSIM: Analysis of similarity
ASVs: Amplicon sequence variants
CCA: Canonical correspondence analysis
cFEC: Cumulative faecal egg count
CSS: Cumulative sum scaling
dpi: Days post-infection
ENA: European Nucleotide Archive
EPG: Eggs per gram
FDR: False discovery rate
FEC: Faecal egg count
GAMM: Generalised additive mixed modelling
GI: Gastrointestinal
LEfSe: Linear discriminant analysis effect size
MELR: Mixed effect linear regression
PCoA: Principal coordinates analysis
PERMANOVA: Permutational multivariate analysis of variance
QIIME2: Quantitative insights into microbial ecology 2
SCFA: Short chain fatty acid
Tci-MEP-1: *Teladorsagia circumcincta* astacin-like metalloprotease
mTci-APY-1: Mutated *Teladorsagia circumcincta* calcium-dependent apyrase
