# Supplementary figures and images for: Vaccination against the brown stomach worm, Teladorsagia circumcincta, followed by parasite challenge, induces inconsistent modifications in gut microbiota composition of lambs
Source: Parasit Vectors. 2021 Apr 6;14:189. doi: 10.1186/s13071-021-04688-4 (PMC8025363; doi:10.1186/s13071-021-04688-4)

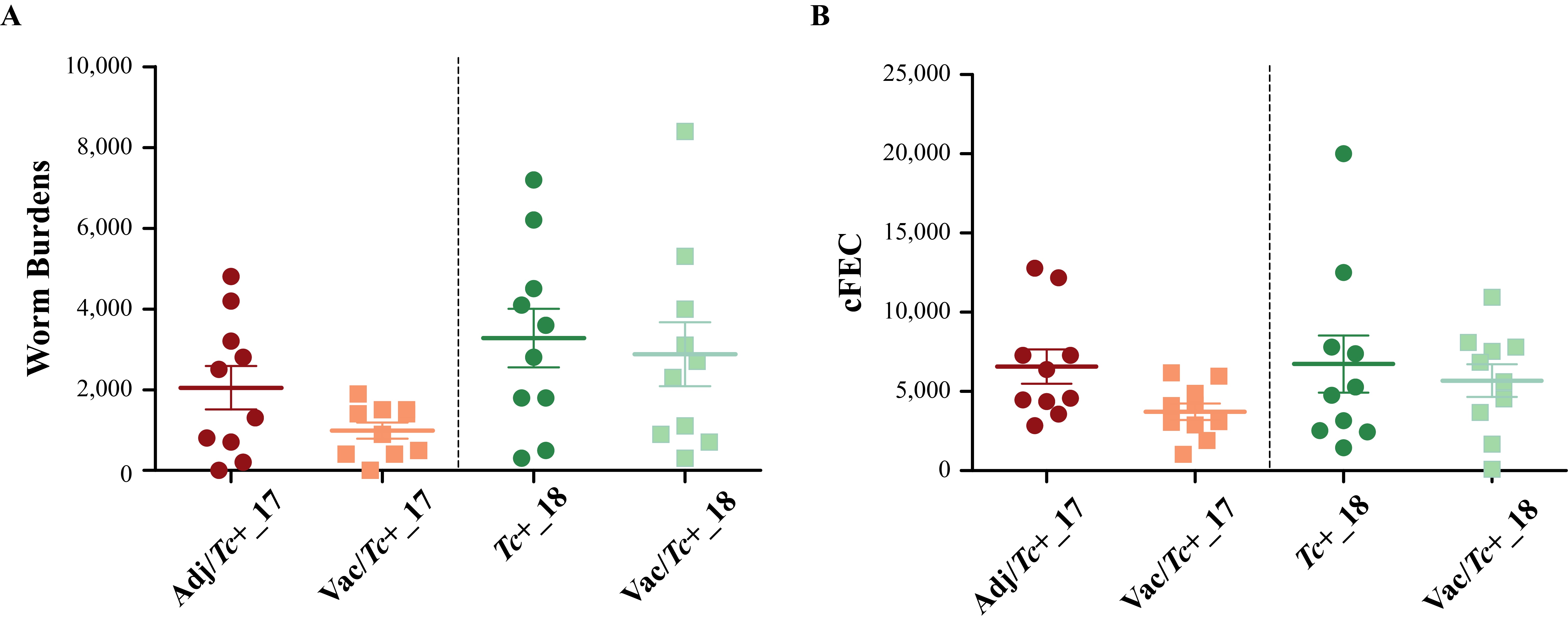

Supplement: Supplementary file 1 — Additional file 1. Mean (± standard error) values of (A) abomasal nematode burdens and cumulative faecal egg counts (cFEC) recovered from lambs infected with Teladorsagia circumcincta over the 2017 and 2018 trials, with (Vac/Tc+_17 and Vac/Tc+_18) or without (Adj/Tc+_17 and Tc+_18) prior immunisation. [file 13071_2021_4688_MOESM1_ESM.jpg]

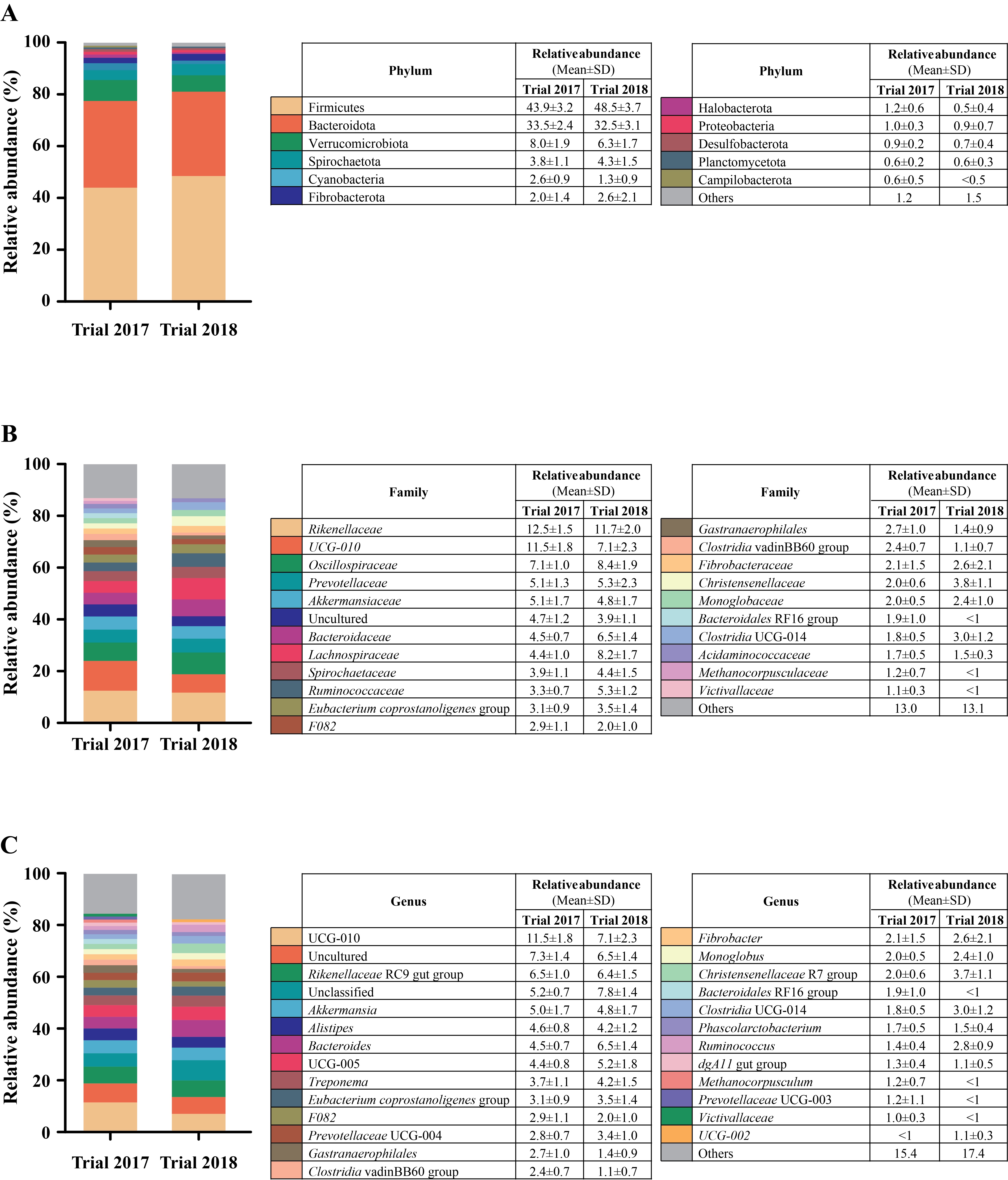

Supplement: Supplementary file 2 — Additional file 2. Gut microbial profiles of lambs enrolled in the 2017 and 2018 trial, respectively, at phylum (A), family (B) and genus (C) level. Plots display the mean relative abundances (calculated by total sum normalisation, i.e. TSS) of each taxon, whilst tables show mean ± standard deviation (SD). ‘Others’ includes all taxa representing < 0.5% (A) or < 1% (B and C) of the whole bacterial population. [file 13071_2021_4688_MOESM2_ESM.jpg]

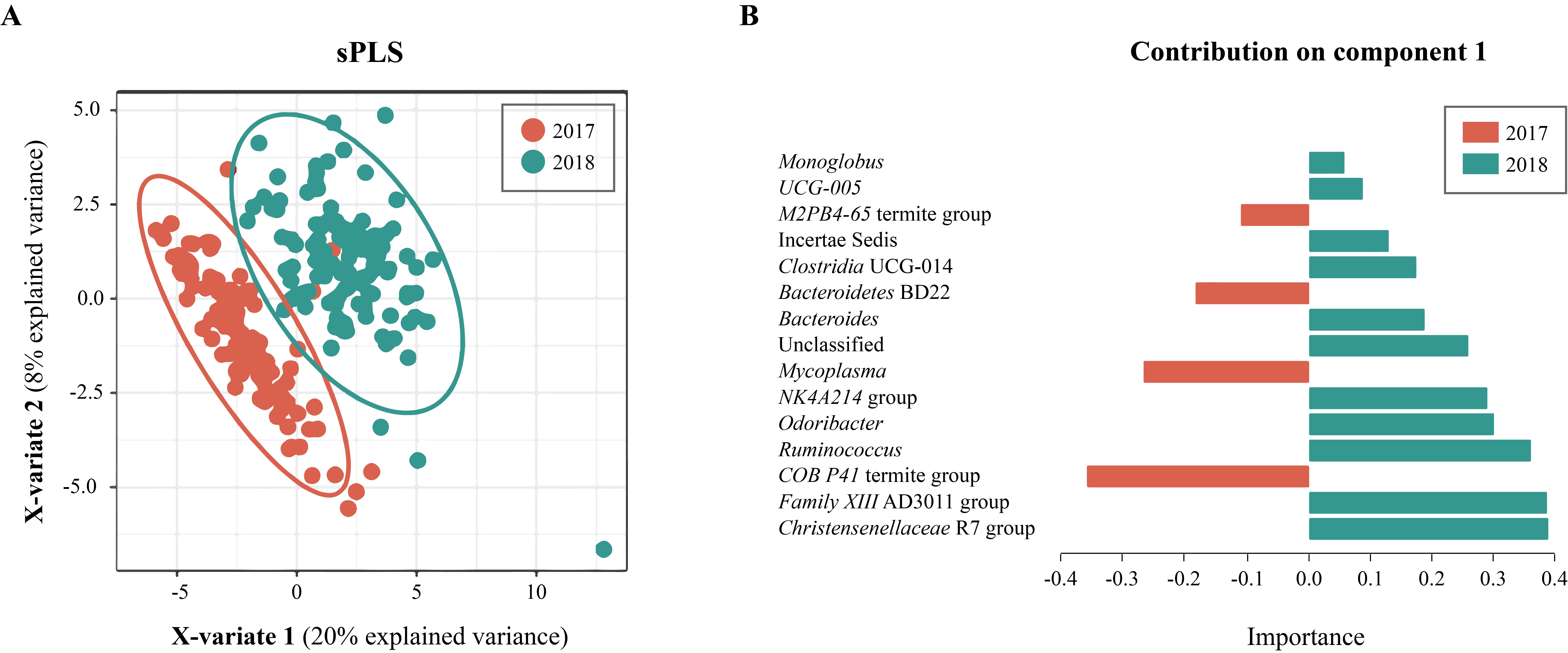

Supplement: Supplementary file 3 — Additional file 3. (A) Sparse Partial Least Squares (sPLS) regression applied to the faecal microbiota of lambs enrolled in each 2017 and 2018 trial. (B) Top bacterial genera discriminating the faecal microbiota of samples collected in each trial, identified by sPLS-Discriminant Analysis (sPLS-DA). [file 13071_2021_4688_MOESM3_ESM.jpg]

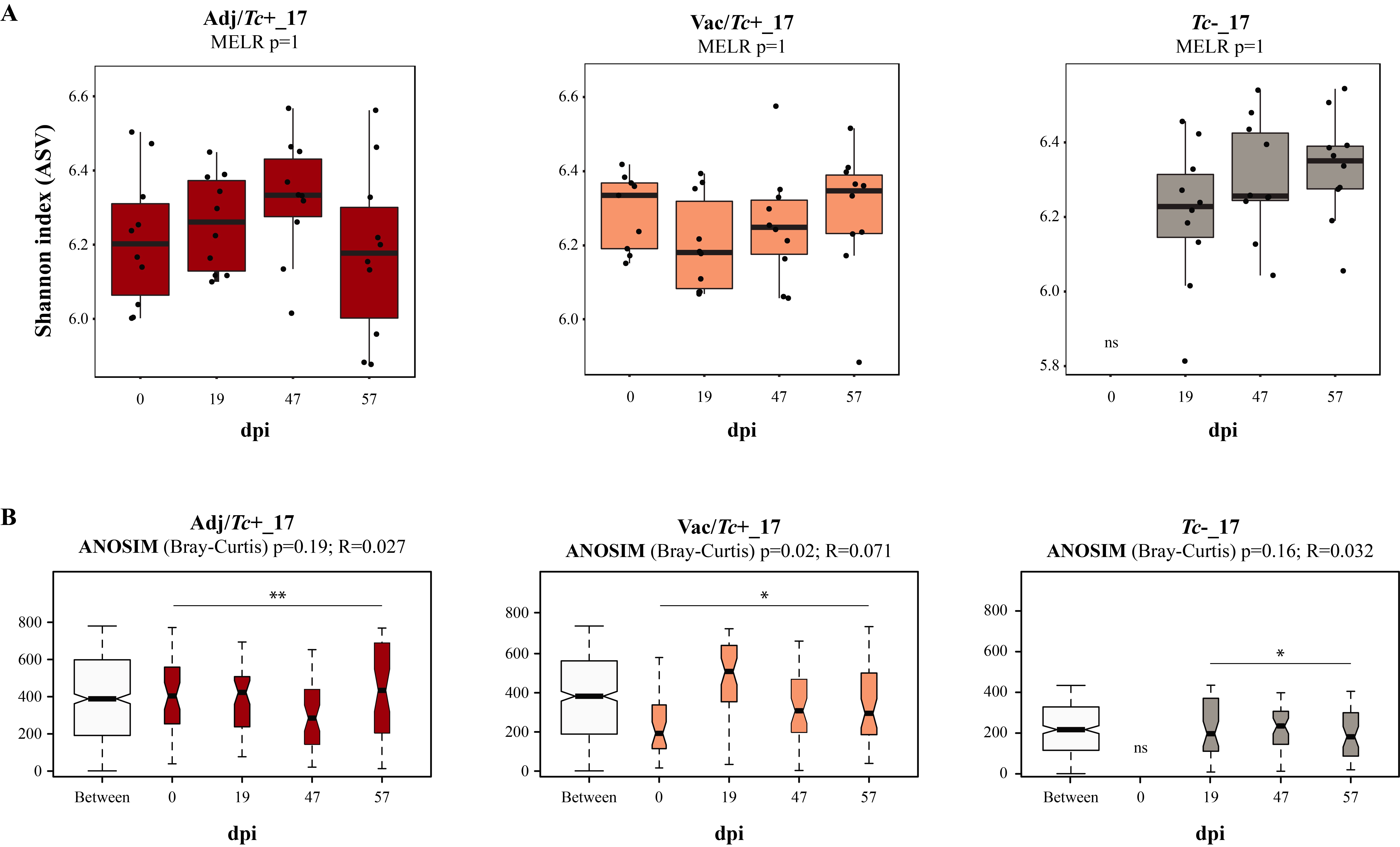

Supplement: Supplementary file 4 — Additional file 4. Longitudinal changes in faecal microbial diversity of lambs experimentally infected with Teladorsagia circumcincta following adjuvant (Adj/Tc+_17) or vaccine (Vac/Tc+_17) administration, and of uninfected controls (Tc-_17), over the course of the 2017 trial. (A) Shannon index for alpha diversity; differences between time points were calculated by Mixed Effect Linear Regression (MELR). (B) ANOSIM plots depicting fluctuations in beta diversity over the course of the trial. Horizontal lines and asterisks indicate statistically significant differences between pairs of time points, calculated by permutational multivariate analysis of variance (PERMANOVA): *q < 0.05; **q < 0.01. ns: no sample available. [file 13071_2021_4688_MOESM4_ESM.jpg]

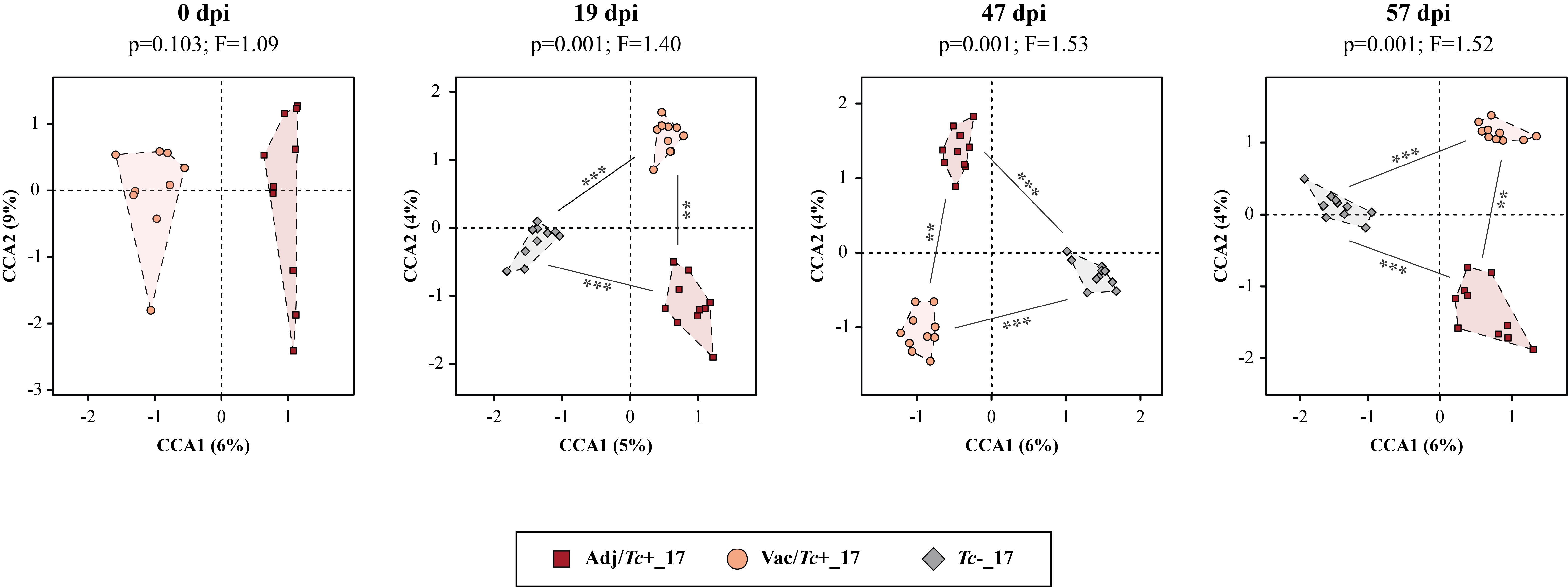

Supplement: Supplementary file 6 — Additional file 6. Faecal microbial profiles of lambs enrolled in the 2017 trial and infected with Teladorsagia circumcincta following adjuvant inoculation (Adj/Tc+_17) or immunisation (Vac/Tc+_17), as well as uninfected controls (Tc-_17), ordinated by Canonical Correspondence Analysis (CCA). Statistical differences between the microbial profiles of each experimental group at each time point post-trickle infection (dpi) are indicated at the top of each plot, whereas asterisks represent statistically significant differences between group pairs: **p < 0.01; ***p < 0.001. [file 13071_2021_4688_MOESM6_ESM.jpg]

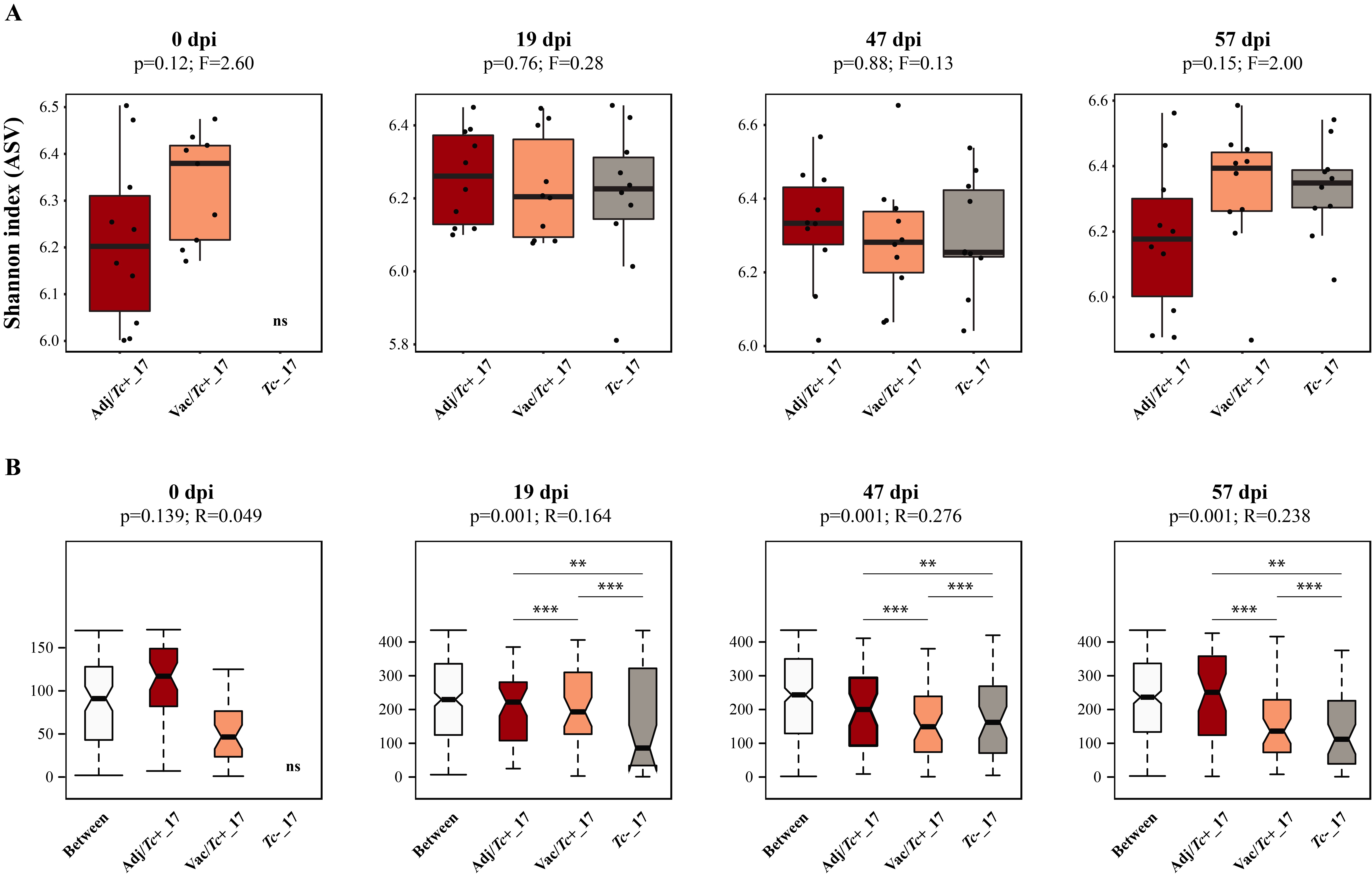

Supplement: Supplementary file 7 — Additional file 7. Differences in microbial alpha (A) and beta (B) diversity between experimental groups of lambs enrolled in the 2017 trial. (A) Shannon index (at Amplicon Sequence Variant level, ASV) calculated for each experimental group at each time point (dpi) and statistical differences between groups were assessed by ANOVA. (B) Overall and pairwise differences in Bray-Curtis dissimilarity between experimental groups were calculated by ANOSIM at each time point: **p < 0.01; ***p < 0.001. Adj/Tc+_17: lambs infected with Teladorsagia circumcincta following inoculation of the vaccine adjuvant; Vac/Tc+_17: lambs immunised against and subsequently experimentally infected with T. circumcincta; Tc-_17: uninfected lambs; ns: no sample available. [file 13071_2021_4688_MOESM7_ESM.jpg]

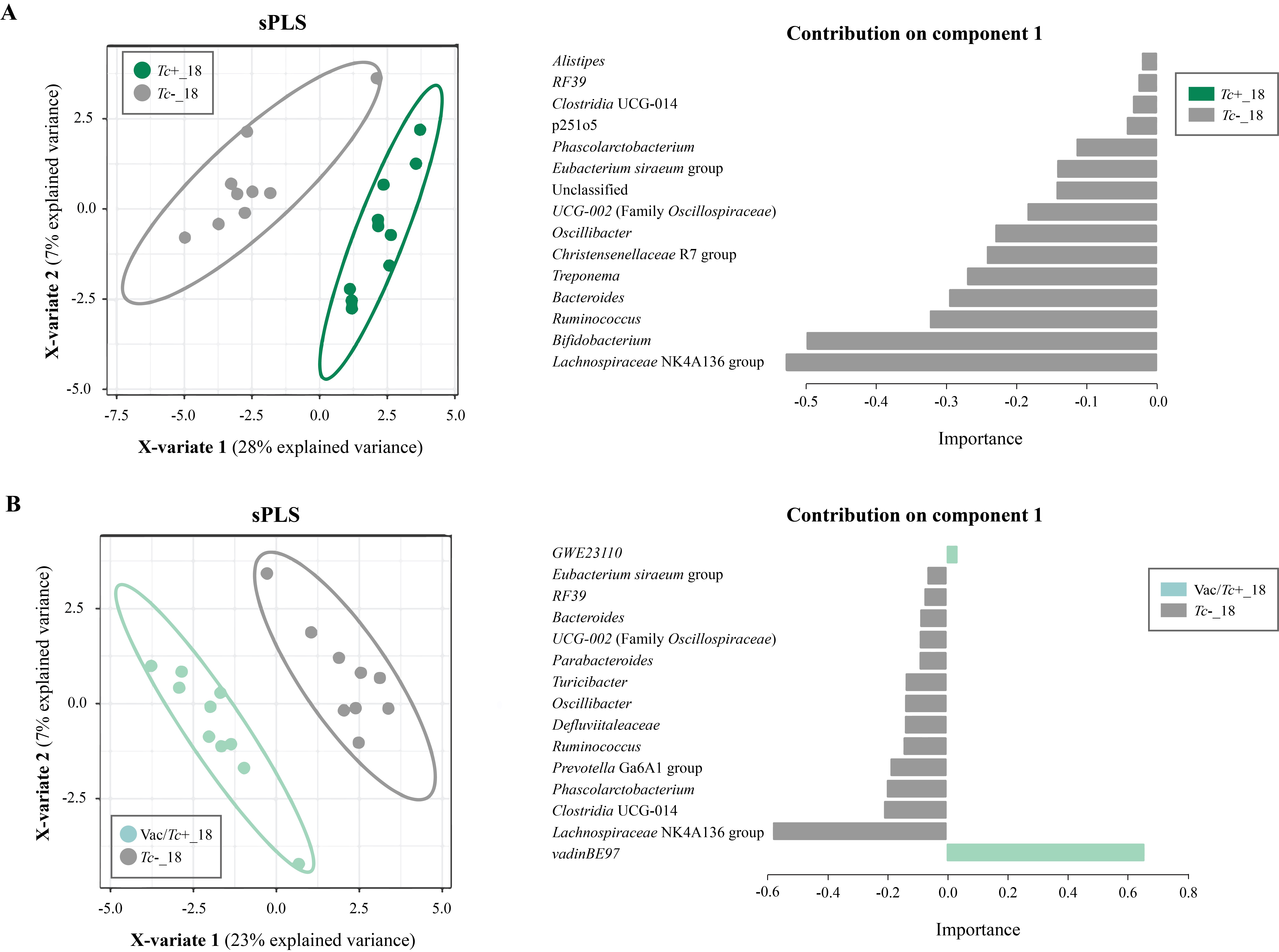

Supplement: Supplementary file 9 — Additional file 9. Sparse Partial Least Squares Discriminant Analysis (sPLS-DA) applied to the faecal microbiota of lambs enrolled in the 2018 trial at 0 days post-trickle infection, clustered by experimental group. (A) Comparison between infected (Tc+_18) vs. uninfected (Tc-_18) animals. (B) Comparison between vaccinated (Vac/Tc+_18) vs. uninfected (Tc-_18) animals. For each group pair compared, sPLS regression (left) and top bacterial genera discriminating the faecal microbiota of samples collected from each experimental animal, identified by sPLS-Discriminant Analysis (sPLS-DA). [file 13071_2021_4688_MOESM9_ESM.jpg]

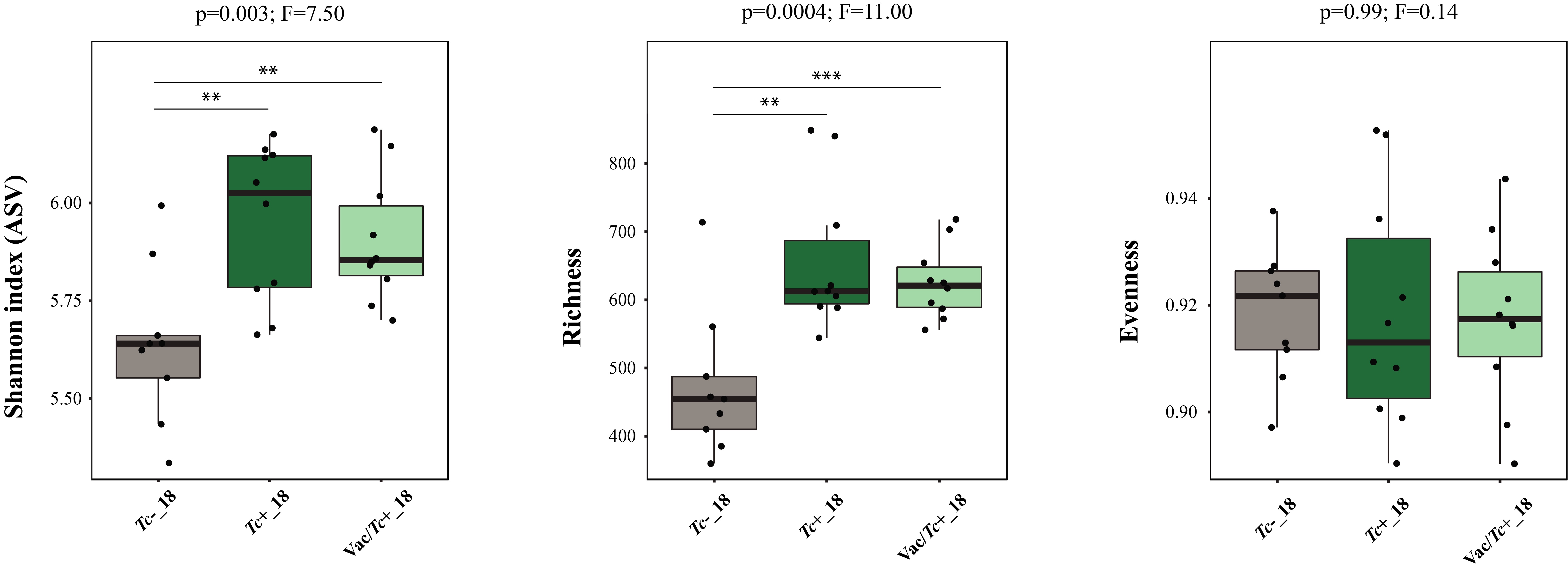

Supplement: Supplementary file 10 — Additional file 10. Differences in microbial alpha diversity between faecal samples of lambs infected with Teladorsagia circumcincta, either with (Vac/Tc+_18) or without prior immunisation (Tc+_18), and of uninfected controls (Tc-_18), enrolled in the 2018 trial. Horizontal lines indicate differences between time points: **p < 0.01; ***p < 0.001. [file 13071_2021_4688_MOESM10_ESM.jpg]

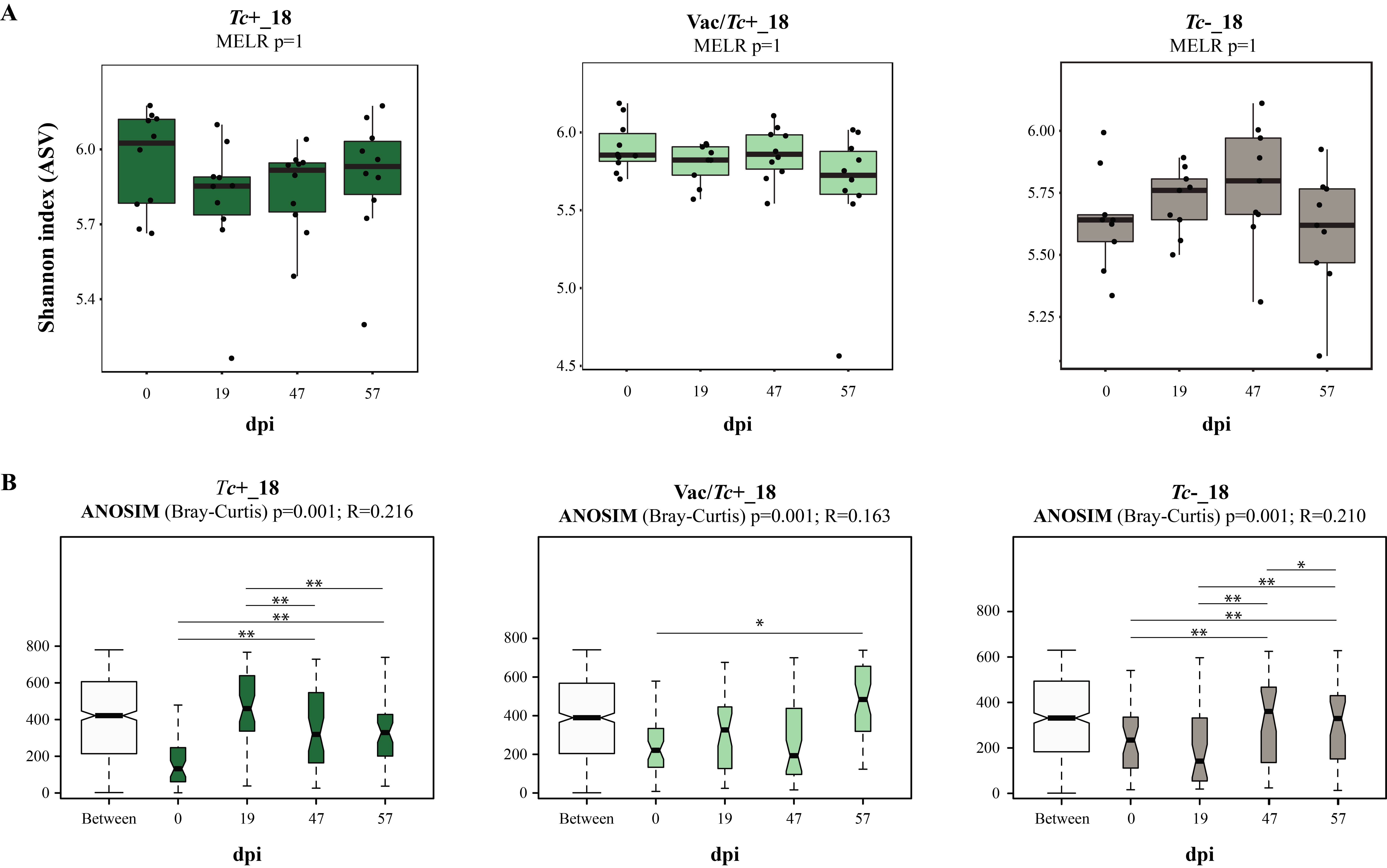

Supplement: Supplementary file 11 — Additional file 11. Longitudinal changes in faecal microbial diversity of lambs enrolled in the 2018 trial and infected with Teladorsagia circumcincta, either with (Vac/Tc+_18) or without prior immunisation (Vac/Tc+_18), and of uninfected controls (Tc-_18). (A) Shannon index for alpha diversity; differences between time points were calculated by Mixed Effect Linear Regression (MELR). (B) ANOSIM plots depicting fluctuations in beta diversity over the course of the trial. Horizontal lines and asterisks indicate statistically significant differences between pairs of time points, calculated by permutational multivariate analysis of variance (PERMANOVA): *q < 0.05; **q< 0.01. [file 13071_2021_4688_MOESM11_ESM.jpg]

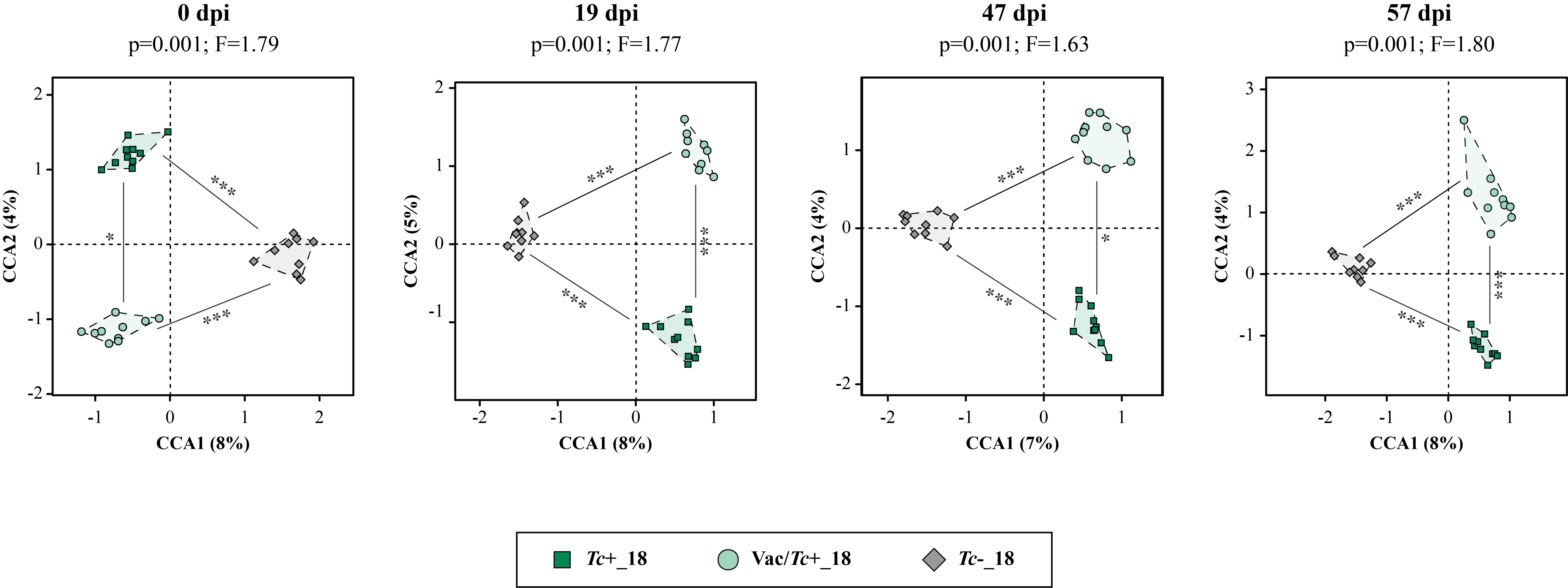

Supplement: Supplementary file 13 — Additional file 13. Faecal microbial profiles of lambs enrolled in the 2018 trial and infected with Teladorsagia circumcincta, either without (Tc+_18) or following prior immunisation (Vac/Tc+_18), as well as uninfected controls (Tc-_18), ordinated by Canonical Correspondence Analysis (CCA). Statistical differences between the microbial profiles of each experimental group at each time point post-trickle infection (dpi) are indicated at the top of each plot, whereas asterisks represent statistically significant differences between group pairs: **p < 0.01; ***p < 0.001. [file 13071_2021_4688_MOESM13_ESM.jpg]

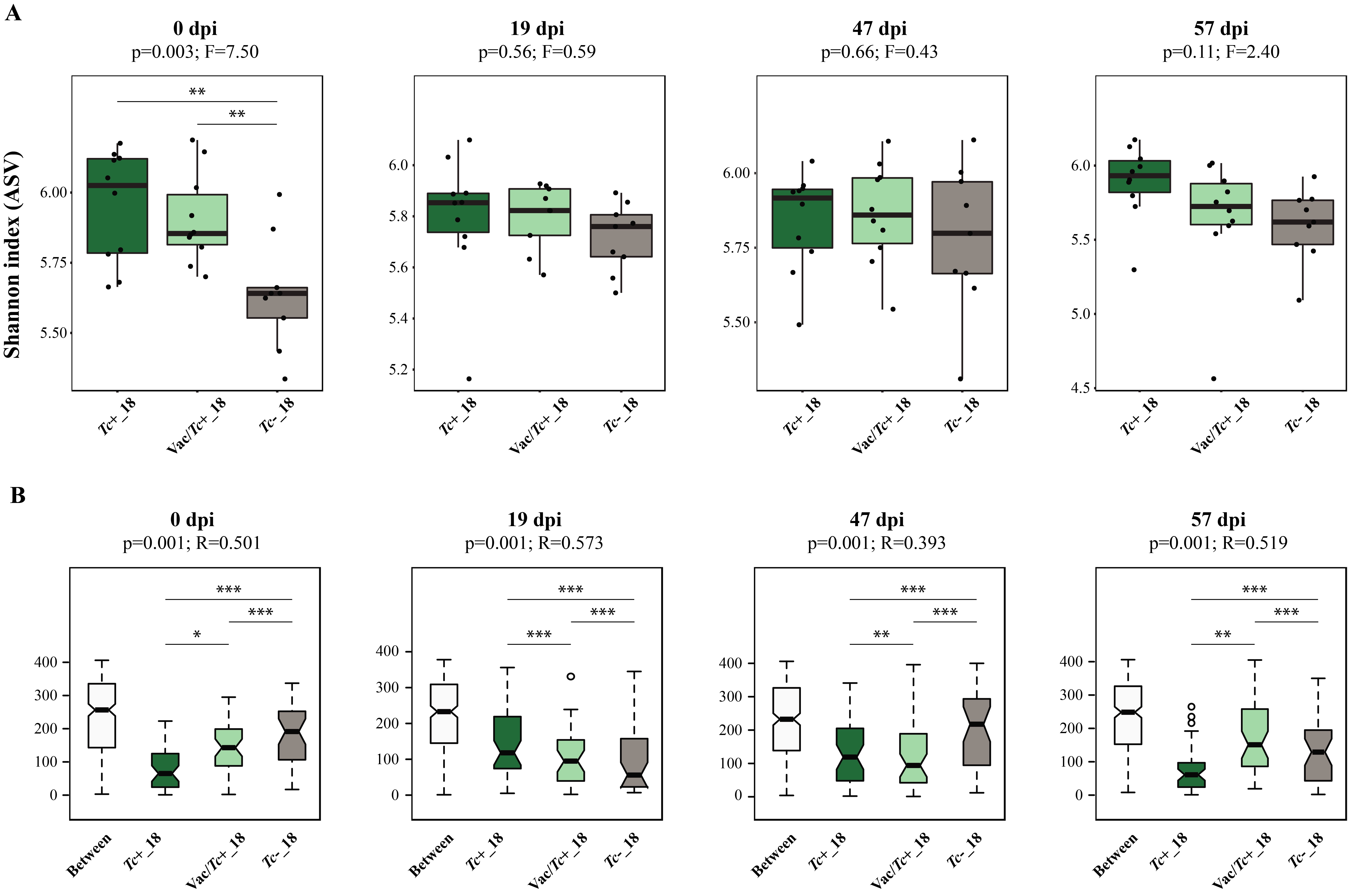

Supplement: Supplementary file 14 — Additional file 14. Differences in faecal microbial alpha (A) and beta (B) diversity between groups of lambs enrolled in the 2018 trial. (A) Shannon index (at Amplicon Sequence Variant level, ASV) calculated for each experimental group at each time point and statistically significant differences between groups assessed by ANOVA. (B) Overall and pairwise differences in Bray-Curtis dissimilarity between experimental groups, calculated by ANOSIM at each time point: *p < 0.05; **p< 0.01; ***p < 0.001. Tc+_18: lambs infected with Teladorsagia circumcincta; Vac/Tc+_18: lambs vaccinated against and subsequently infected with T. circumcincta; Tc-_2018: uninfected lambs. [file 13071_2021_4688_MOESM14_ESM.jpg]
